# Estimation of rice yield using multivariate analysis techniques based on meteorological parameters

**DOI:** 10.1038/s41598-024-63596-6

**Published:** 2024-06-01

**Authors:** Ajay Sharma, Joginder Kumar, Mandeep Redhu, Parveen Kumar, Mohit Godara, Pushpa Ghiyal, Pingping Fu, Mehdi Rahimi

**Affiliations:** 1 Department of Mathematics and Statistics, CCS, Haryana Agricultural University, Hisar, Haryana India; 2 Depaprtment of Plant, Soil and Agricultural System, Southern Illinois University, Carbondale, IL USA; 3 Department of Agronomy, CCS, Haryana Agricultural University, Hisar, Haryana India; 4 Department of Agricultural Meteorology, CCS, Haryana Agricultural University, Hisar, Haryana India; 5 School of Education, Southern Illinois University, Carbondale, IL USA; 6 Department of Biotechnology, Institute of Science and High Technology and Environmental Sciences, Graduate University of Advanced Technology, Kerman, Iran

**Keywords:** Rice yield, Estimation, Weather parameters, Multivariate analysis, RMSE and MAPE, Ecology, Plant sciences, Climate sciences

## Abstract

This study aims to develop predictive models for rice yield by applying multivariate techniques. It utilizes stepwise multiple regression, discriminant function analysis and logistic regression techniques to forecast crop yield in specific districts of Haryana. The time series data on rice crop have been divided into two and three classes based on crop yield. The yearly time series data of rice yield from 1980–81 to 2020–21 have been taken from various issues of Statistical Abstracts of Haryana. The study also utilized fortnightly meteorological data sourced from the Agrometeorology Department of CCS HAU, India. For comparing various predictive models' performance, evaluation of measures like Root Mean Square Error, Predicted Error Sum of Squares, Mean Absolute Deviation and Mean Absolute Percentage Error have been used. Results of the study indicated that discriminant function analysis emerged as the most effective to predict the rice yield accurately as compared to logistic regression. Importantly, the research highlighted that the optimum time for forecasting the rice yield is 1 month prior to the crops harvesting, offering valuable insight for agricultural planning and decision-making. This approach demonstrates the fusion of weather data and advanced statistical techniques, showcasing the potential for more precise and informed agricultural practices.

## Introduction

Rice is the most significant and widely planted crop in India and in terms of global rice output, India comes at second place. The north-eastern, southern, and south-eastern regions of India account for 92% of the country's rice output. Approximately, 44 million hectares of land in India is used for rice farming^1^. The country's rice production is influenced by monsoon patterns, irrigation facilities, government policies, and market demand. States like Punjab, West Bengal, Uttar Pradesh, Andhra Pradesh, and Telangana are significant contributors to India's rice production. Haryana is a major agricultural state in India, known for its wheat and rice production. The state has made significant strides in agricultural technology, irrigation facilities, and crop management practices. Haryana's rice production is supported by the availability of water resources from rivers like the Yamuna and the infrastructure for rice cultivation^2^.

Weather variables can have differing effects on crops at various stages of development. Therefore, the influence of weather on crop yield not only depends on the intensity of weather variables but also on their distribution pattern throughout the crop season. This highlights the need to divide the entire crop season into smaller intervals and analysis crop-weather relationships within these intervals. However, this approach increases the number of variables in the model, leading to a large number of parameters that need evaluation from the available data. Due to limited data availability, it may be challenging to precisely estimate these parameters. Thus, a technique that uses a manageable number of parameters while considering the entire weather distribution can be a viable solution to address this issue.

India possesses one of the world's most effective systems for gathering, organizing, and summarizing data on crop production. This system relies on official inputs regarding area and yield obtained from states, which in turn collect data from districts and further sources. Area data is derived from comprehensive assessments conducted by revenue agencies, while yield data comes from crop cutting experiments. The Directorate of Economics and Statistics, Ministry of Agriculture in New Delhi issues initial forecasts (advance estimates) for major cereal and commercial crops. However, the final estimates are typically provided several months after the actual crop harvest. As a result, one drawback of the Department of Agriculture's yield estimates is the delay and potential quality issues with the statistics. Therefore, there is significant room for enhancing the conventional system.

Climate change has become a significant concern, prompting researchers to delve into its effects on crop growth and yield. They are also focused on identifying appropriate management strategies to maintain crop productivity in the face of projected climate changes. To achieve a quantitative understanding of how crops are response to climate shifts, researchers are developing statistical models that consider the crop's time-series behaviour along with various climatic factors. Summer crops are particularly vulnerable to low temperatures during reproductive stages, and their differing responses to temperature decrease can significantly impact crop yields. The challenge lies in integrating this relevant information into the forecasting process and subsequently into decision-making processes and for an operational yield model to gain widespread adoption, it's crucial to have access to data well before the crop harvest, and the data collection process should be cost-effective. Weather parameters are readily available during the maximum vegetative stage of the crop and can be highly effective for developing accurate yield forecasting models.

Therefore, it becomes essential to predict the rice yield in advance. The interplay between climatic factors and the crop's growth stages significantly influences both the total yield and the ability to forecast it before harvesting. Accurate predictions are invaluable, not just for farmers but also for trade, industry, and policymakers. Projections of rice yield play a crucial role for taking decisions related to pricing, storage, marketing strategies, and distribution channels, emphasizing their substantial impacts across the agricultural sector and beyond. In past years, many researchers have been tried different techniques to develop rice yield forecasting models such as models (weather indices)^3^, artificial neural network^4^, principal component analysis^5^ and time series^6^.

Other than these techniques, forecasting of rice yield using different statistical technique such as ordinal logistic regression and discriminant function analysis have been discussed in past by several researchers including Goyal^7^ studied several statistical techniques for estimating wheat yield such as multiple linear regression, discriminant analysis, and principal component analysis etc. Sharma et al.^8^ studied the estimation of wheat yield based on environmental factors using ordinal logistic regression. Kumari and Kumar^9^ used ordinal logistic regression for forecasting the yield. Kumari et al.^10^ explored the crop production output in Kanpur district of Uttar Pradesh by utilizing logistic regression to predict future yield. Kumar et al.^11^ developed pre-harvest forecast models of yield using advance statistical technique based on meteorological parameters. Bayesian discriminant and discriminant function analysis (scores) both the technique have been contrasted by Kumari et al.^12^. Goyal and Verma^13^ used various statistical techniques for pre-harvest crop yield estimation. Priya et al.^14^ utilized discriminant function analysis to predict the yield of Coimbatore in Tamil Nadu by considering by various weather parameters^14^. Discriminant function analysis was performed to forecast the sugarcane yield in Coimbatore district, Tamil Nadu by using monthly weather data and the yield was categorized into two and three groups. The scores derived from this analysis, along with the trend, was incorporated as regressors in developing the yield forecast models. Comparisons between the forecasting models based on two groups and three groups indicated that the models utilizing three groups were deemed more effective. Given the information provided, the current research aims to create predictive models for rice yield before harvesting by operating weather data and advanced statistical methods.

## Materials and methods

### Area and crop covered

The research was conducted in Karnal district, Haryana, India, located at coordinates approximately 29.68570 N latitude and 76.99050 E longitude, situated within the eastern plain zone of Haryana. With an average annual rainfall of around 766 mm and a soil composition primarily consisting of deep alluvial, medium to medium-heavy textured soils that are easy to plough, the area offers favourable conditions for agriculture. The combination of favourable climate, soil, and extensive irrigation facilities makes rice and wheat cultivation a natural choice for the region. Rice is typically cultivated during the kharif season, while wheat is grown during the Rabi season, taking advantage of the optimal growing conditions during these periods.

### Data description

The various issues of Statistical Abstract of Haryana have been used to extract the time series data from 2017–18 to 2020–21 on rice crop yield for the Karnal district of Haryana. The study gathered daily meteorological information from the Meteorology Department at C.C.S Haryana Agricultural University in Hisar, Haryana, as well as from CSSRI in Karnal.

### Computation of weather parameters

The weather parameters including maximum temperature (°C), minimum temperature (°C), average relative humidity (%), sunlight hours (h) and cumulative rainfall (mm) are the significant weather parameters which influenced the crop growth, different physiological stages and the rate of phenological development. The fortnight climate information was mentioned below:

$${\text{Average maximum temperature }}\left( {{\text{TMAX}}} \right) \, = \frac{{\mathop \sum \nolimits_{i = 1}^{15} TMAX_{i} }}{15}$$

$${\text{Average minimum temperature }}\left( {{\text{TMIN}}} \right) \, = \frac{{\mathop \sum \nolimits_{j = 1}^{15} TMIN_{j} }}{15}$$

$${\text{Average relative humidity }}\left( {{\text{ARH}}} \right) \, = \frac{{\mathop \sum \nolimits_{k = 1}^{15} TMIN_{k} }}{15}$$

$${\text{Average sunshine hours }}\left( {{\text{SSH}}} \right) \, = \frac{{\mathop \sum \nolimits_{l = 1}^{15} SSH_{l} }}{15}$$

$${\text{Accumulated rainfall }}\left( {{\text{ARF}}} \right) \, = \mathop \sum \limits_{m = 1}^{15} ARF_{m}$$

where TMAX_i_ = $i$th day maximum temperature; TMIN_j_ = $j$th day minimum temperature; ARH_k_ = $k$th day relative humidity; SSH$_{l}$ = $l$th day sun shine hours; ARF_m_ = $m$th day rainfall; (I, j, k,$l^{ }$, m, represent daily weather data).

These data were organized into different fortnight periods based on SMW (Standard Meteorological Weeks) relevant to the growth stages of the rice crop.

### Methodology used for the study

**Figure 1:**
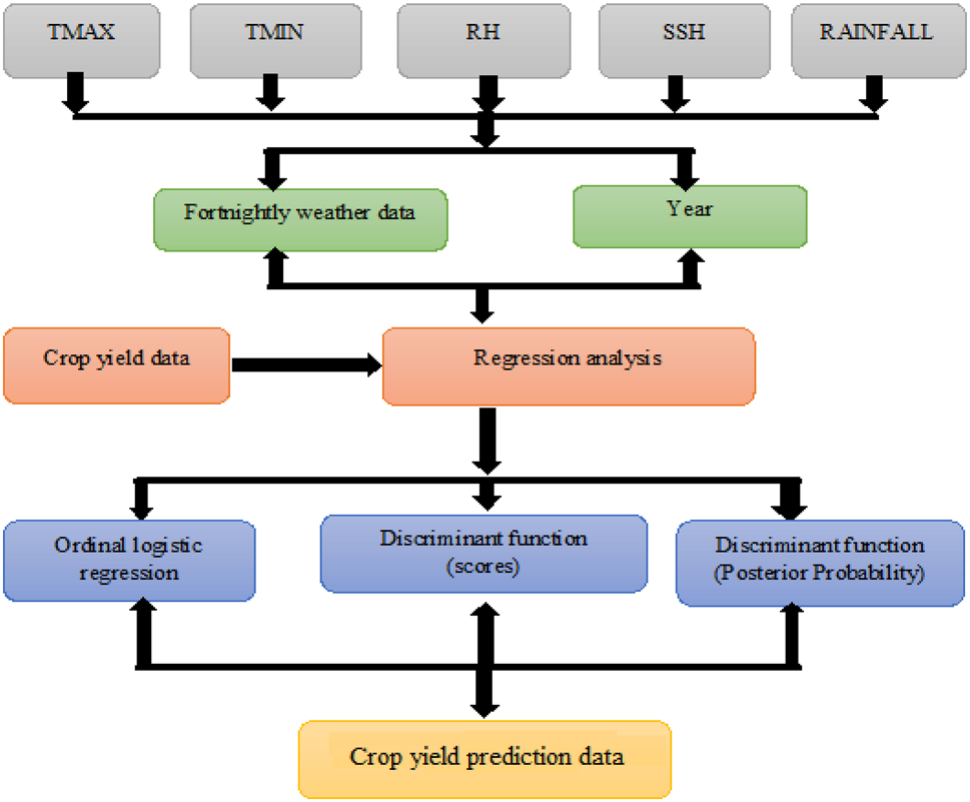
Flowchart of model preparation.

The Fig. 1 illustrates the stages involved in preparing the model.

The forecasting models were fitted using data from 1980–81 to 2017–18 and the next 3 years i.e., from 2018–19 to 2020–21 were used to validate the developed models. Initially, a linear regression analysis was conducted between rice yield (as the response variable) and year (as the explanatory variable), using yield data from 1980–81 to 2017–18. The equation derived from this analysis was then used to calculate the residuals. The residuals were then used to classify the crop yield into two separate categories or groups. The first group was based on two types of residuals: assigning a zero to negative residuals and a one to positive residuals. In the second group, three categories of residuals were organized in ascending order. Subsequently, the crop yield was divided into three classifications: adverse (group 0), normal (group 1), and congenial (group 3). Different statistical methodologies, such as logistic regression and discriminant function analysis, were applied to these two and three groups to determine probabilities and scores for the yield forecasting models. Furthermore, a stepwise linear regression method was utilized to develop these forecast models using the derived probabilities/scores and years as regressors. The effectiveness and accuracy of the fitted models were then assessed by comparing various key measures including the mean absolute percentage error (MAPE), root mean square error (RMSE), mean absolute deviation (MAD), and predicted error sum of squares (PRESS). These measures were critical in validating the reliability and performance of the fitted models.

### Forecast models

The techniques of discriminant function analysis and logistic regression were applied to rice yield forecasting. Regression models based on logistic probabilities and discriminant function analysis based on scores along with trend (year) as regressors were fitted for quantitative rice yield forecasting Kumari and Kumar^9^.

### Two group method

#### Discriminant scores

$${\text{Yield }} = \alpha_{0} + \beta_{1} z_{ } + \beta_{2} T + \varepsilon$$

where $\alpha_{0}$ represented the intercept of the equation; T denoted the the time period, measured in years; $\beta_{i}{\prime} s$ were the coefficients in the regression equation; Z referred to the discriminant score; is error ~ N (0, σ^2^): ε symbolized the error term, assumed to be normally distributed with a mean of 0 and a variance of $\sigma^{2}$.

#### Ordinal logistic regression

The explanatory variable X and the response variable Y had the following linear relationship:

$${\text{Y}} \, = \alpha + \beta {\text{X}} + \varepsilon$$

where $\alpha$ represented the intercept of the equation; $\beta$ was the regression coefficient, denoted the degree of change in Y for a unit change in x; $\varepsilon$ ~ N (0, $\sigma$^2^).

The model was given by

$${\text{Yield }} = \beta_{0} + \beta_{1} P_{1} + \beta_{2} T + \varepsilon$$

where β_0_ was the intercept of the equation; T represented the time period, typically expressed in years. P_1_ denoted the probability of the response variable Y being equal to 1. β_i_’s were the regression coefficients, indicating the influence of each explanatory variable on the response variable. ε represented the error term.

### Three groups

#### Discriminant function analysis (scores)

$${\text{Yield }} = \beta_{0} + \beta_{1} z_{1} + \beta_{2} z_{2} + \beta_{3} T + \varepsilon$$

where z_1_ and z_2_ represent discriminant scores and the other terms retained their previously defined meanings.

#### Ordinal logistic regression

$${\text{Yield }} = \beta_{0} + \beta_{1} P + \beta_{2} P_{2} + \beta_{3} T + \varepsilon$$

where P_1_ and P_2_ are probabilities of Y = 1 and Y = 2 and the other terms retained their previously defined meanings.

#### Comparative performance measures

The performance of a model in predicting the rice yield, or any other variable for that matter, was typically evaluated using various metrics that assessed its accuracy, reliability, and generalizability. The standard metrics often employed for assessing model performance include mean absolute percentage error (MAPE), root mean square error (RMSE), mean absolute deviation (MAD), and predicted error sum of squares (PRESS). These metrics offered valuable insights into the accuracy and reliability of a model's predictions, allowing for a comprehensive evaluation of its performance Kumar et al.^11^.

- (a) Predicted error sum of square (PRESS)

The PRESS statistic was defined as

$${\text{PRESS}} = \mathop \sum \limits_{{{\varvec{i}} = 1}}^{{\varvec{n}}} [{\varvec{Y}}_{{\varvec{i}}} - \hat{\user2{Y}}_{{\user2{i }}} ]^{2}$$

where $Y_{i}$ was the value of dependent variable of ith observation such that rice yield of ith year and $\hat{Y}_{i}$ was the forecast of $Y_{i}$ computed from fitted model without taking the ith data point. It is generally regarded as how well for a model will perform in predicting the rice yield.

- (b) Root mean square error of forecasts
  $${\text{RMSE }} = \sqrt {\frac{1}{{\varvec{n}}}\mathop \sum \limits_{{{\varvec{i}} = 1}}^{{\varvec{n}}} \left( {{\varvec{Y}}_{{\varvec{i}}} - \hat{\user2{Y}}_{{\user2{i }}} } \right)\user2{ }^{2} }$$

where $Y_{i }$ and $\hat{Y}_{i }$ were the observed and forecasted values of the rice yield respectively and n was the number of years for which forecasting was done.

- (c) Mean absolute percentage error of forecast

The formula of MAPE is given as: .

$${\text{MAPE }} = \frac{100}{{\varvec{n}}}\mathop \sum \limits_{{{\varvec{i}} = 1}}^{{\varvec{n}}} |\frac{{\hat{\user2{Y}}_{{\user2{i }}} - {\varvec{Y}}_{{\varvec{i}}} }}{{\user2{ Y}_{{\varvec{i}}} }}|$$

where $Y_{i }$ and $\hat{Y}_{i }$ were the observed and forecasted value of the rice yield respectively and n was the number of years for which forecasting was done.

- (d) Mean absolute deviation

The formula of MAD is given as:

$${\text{MAD }} = \frac{1}{{\varvec{n}}}\mathop \sum \limits_{{{\varvec{i}} = 1}}^{{\varvec{n}}} \user2{ }\left| {{\varvec{Y}}_{{\varvec{i}}} - \hat{\user2{Y}}_{{\user2{i }}} } \right|$$

where ${\text{Y}}_{i}$ and ${\hat{\text{Y}}}_{i}$ were the observed and forecasted value of the rice yield respectively and n was the number of years for which forecasting was done.

## Results and discussion

It sounds like an extensive analysis was conducted to develop the forecasting models using various statistical procedures. Each of these methods had its own strengths and considerations viz., multiple linear regression, stepwise regression, ordinal logistic regression and discriminant function analysis. By employing these diverse statistical procedures, the analysis attempted to capture different aspects of the relationship between weather patterns and rice crop yield. Each method might emphasize certain aspects or patterns within the data, offering unique insights into how weather variables impact the yield.

### Forecast models

The stepwise linear regression technique was utilized to fit regression models, incorporating probabilities derived from ordinal logistic regression (OLR), discriminant function analysis^15^, and the year as independent variables, with yield as the dependent variable. The resulting rice yield forecast models for different fortnights were detailed in Table 1.

**Table 1:** Rice yield forecast models using OLR and DFA based on scores for different fortnights.

Fortnight (SMW)	Stepwise regression equation	Adjusted R^2^
Two groups		
Discriminant model		
21st fortnight	Y = − 464.684 + 0.253 T + 0.937Z (0.041) (0.264)	0.677
22nd fortnight	Y = − 358.116 + 0.184 T + 0.788Z (0.062) (0.265)	0.640
23rd fortnight	Y = − 353.028 + 0. 194 T + 0.793Z (0.079) (0.291)	0.653
Logistic model		
21st fortnight	Y = − 480.774 + 0.261 T + 3.567P (0.044) (1.196)	0.647
22nd fortnight	Y = − 359.940 + 0.186 T + 2.843P (0.065) (1.145)	0.614
23rd fortnight	Y = − 350.674 + 0.196 T + 2.933P (0.080) (1.196)	0.639
Three groups		
Discriminant model		
21st fortnight	Y = − 415.651 + 0.226t + 0.415Z_1_ + 1.096Z_2_ (0.039) (0.211) (0.311)	0.730
22nd fortnight	Y =—316.091 + 0.166 T + 0.380Z_1_ + 1.118Z_2_ (0.054) (0.201) (0.268)	0.719
23rd fortnight	Y = − 449.461 + 0.244 T + 0.391Z_1_ + 1.026Z_2_ (0.071) (0.207) (0.289)	0.713
Logistic model		
21st fortnight	Y = − 762.331 + 0.401 T − 3.124P_1_ − 6.951P_2_ (0.605) (1.983) (1.836)	0.693
22nd fortnight	Y = − 628.011 + 0.319 T − 2.898P_1_ − 6.317P_2_ (0.083) (1.960) (2.002)	0.682
23rd fortnight	Y = − 719.513 + 0.380 T − 3.787P_1_ − 6.522P_2_ (0.089) (1.757) (2.056)	0.653

Figures in brackets represent standard errors.

**Significant at 1% level of significance.

*Significant at 5% level of significance.

### Selected forecast models

The rice yield was divided into two groups: negative residuals representing low yield (0), and positive residuals representing high yield (1). The model used Probability (P) and score (Z) as predictors, and the outcomes, along with adjusted R^2^ values, were presented in Table 2. When considering three yield groups, 40 years of yield data were sorted into low (0), medium (1), and high (2) categories based on residuals. The same methodology was applied, but with the addition of two probabilities (P1 and P2) and scores (Z1 and Z2) within each group.

**Table 2:** Selected regression models (two group).

Stepwise linear regression based on	Equations of selected model	Adjusted R^2^
Discriminant scores (21st fortnight)	Y = − 464.684 + 0.937Z** + 0.253 T** (0.264) (0.041)	0.677
Logistic probabilities (21st fortnight)	Y = − 480.774 + 3.567P* + 0.261 T* (1.196) (0.044)	0.647

Figures in brackets represent standard errors.

**Significant at 1% level of significance.

*Significant at 5% level of significance.

In Table 2, the selected models using stepwise linear regression by taking discriminant scores (Z), logistic probabilities(P) and time (T) as regressors were given. Also, it can be seen that the value of coefficient of determination is 67.7% in case of discriminant scores and 64.7% in case of logistic regression models for forcasting the rice yield.

The above Table 3, showed developed models using stepwise linear regression by taking discriminant scores (Z_1_ and Z_2_), logistic probabilities (P_1_andP_2_) and year (T) as regressors.

**Table 3:** Selected equation models (three group).

Stepwise regression models based on	Equations of selected model	Adjusted R^2^
Discriminant scores (21st fortnight)	Y = − 415.651 + 0.415Z_1_** + 1.096Z_2_** + 0.226 T** (0.211) (0.311) (0.039)	0.730
Logistic probabilities (21^st^ fortnight)	Y = − 762.331–3.124P_1_* − 6.951P_2_* + 0.401 T** (1.983) (1.836) (0.605)	0.693

Figures in b rackets represent standard errors.

**Significant at 1% level of significance.

*Significant at 5% level of significance.

**Figure 2:**
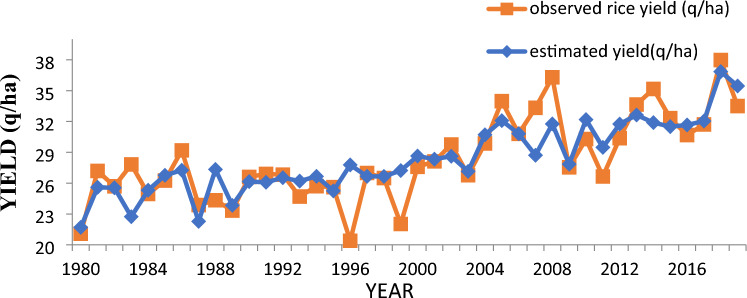
Observed and predicted rice yield for selected model.

In Table 4, the values of performance measures like PRESS, RMSE, MAPE and MAD for three groups based on discriminant scores were found lowest as compared to other models. In Fig. 2, the values of observed and predicted yield using graphical representation were given to provide a clear visual comparison of the selected models. Also, it can be seen clearly from the below figure that the yield was sudden decreased in the year 1996 and 1999. This yield gap may occur when irrigated rice under tropical climates and sudden increase in 2007–2008 may due to favourable conditions as compared to other year^16^. It is also found that the average absolute relative deviation (%) for the training set was 28.08 (%) approximately and in case of selected model i.e. discriminant function analysis based on scores is 3.37 (%).

**Table 4:** Comparison of logistic regression and discriminant function analysis (scores).

Accuracy measures	Discriminant		Logistic regression	
	Two group	Three group	Two group	Three group
PRESS	19.52	17.06	19.84	46.34
RMSE	2.55	2.38	2.57	3.93
MAPE	4.10	3.56	5.01	8.24
MAD	1.38	1.36	1.91	3.12

## Discussion

In the course of presenting the results of the present study, the significant variations were observed due to the effect of different weather parameters on the rice yield. The study emphasized the importance of timely and reliable crop forecasts for an agrarian economy, which are essential for planning, policy formulation, and implementation related to crop procurement, price structure, distribution, and import–export decisions. The study included fitting of stepwise linear regression models with yield as the response variable and weather parameters and year as regressors, as well as studying ordinal logistic regression and discriminant function analysis for forecasting the crop yield based on weather variables. The present study was designed to develop models for estimating rice yield using ordinal logistic regression and discriminant function analysis. The objectives included creating a yield forecast model for rice crop using ordinal logistic regression with weather data and comparing the performance of ordinal logistic regression and discriminant function analysis. Johnson et al.^17^ conducted a study on the relationship between weather conditions and outbreaks of potato late-blight in the semiarid region of south-central Washington. They used linear discriminant analysis and logistic regression analysis to forecast late-blight outbreaks based on weather variables. Their findings revealed that the logistic regression model outperformed the discriminant function analysis in predicting late-blight outbreaks. Hassan et al.^18^ said that the discriminant analysis and binary logistic regression enabled more accurate prediction of autism spectrum disorder than principal component analysis^19^. Multivariate analysis was utilized to identify the key distinguishing factors between COVID-19 patients and healthy individuals, as well as between severe and moderate cases. By employing discriminant analysis and binary logistic regression models, we achieved a classification accuracy ranging from 71 to 100%. The differentiation of severe cases from moderate ones was primarily associated with reduced levels of natural killer cells and activated class-switched memory B cells, higher neutrophil frequency, and decreased HLA-DR expression on monocytes in severe COVID-19 patients. Garde et al.^20^ studied the different statistical models based on weather parameters and also developed statistical model using data from 1990 to 2012, and validation was carried out using the remaining data from 2013 to 2016. The adjusted R^2^ values ranged from 73.00 to 93.30% across different models. The best forecast model was selected based on high adjusted R^2^ values, forecast error, and RMSE. In Navsari district, the discriminant function analysis technique (Model-5) was found to be superior to logistic regression analysis (Model-12) for pre-harvest forecasting of rice crop yield, based on the obtained results. The present study involved using stepwise linear regression, ordinal logistic regression, and discriminant function analysis based on scores for forecasting the rice yield. The detrended yield is divided into two and three categories, with years and weather variables considered as regressors. The study compares the accuracy of the fitted models using PRESS, RMSE, MAPE and MAD. In the case of two categories, discriminant function analysis (scores) yielded optimal results, while in the case of three categories, discriminant function analysis (scores) also performed better compared to the ordinal logistic regression method. The best time for forecasting rice yield is found to be 1 month before harvesting i.e. (21st Fortnight).

## Conclusion

In this study, we explored the prediction of rice yield using different statistical methods such as regression analysis, ordinal logistic regression, and discriminant function analysis and highlighted their unique roles and importance in forecasting the rice yield. Each approach played a crucial part in understanding, modeling, and categorizing yield data. Several multivariate models were fitted using fortnightly weather data by categorizing the yield data into two and three groups. The performance of these selected models were compared using various performance measures such as PRESS, MAPE, RMSE, and MAD to assess their effectiveness. According the findings of present study, the discriminant function analysis based on scores provided the optimum results as compared to other methods. Hence, it is concluded that the discriminant function analysis(scores) in case of three groups performed best as compared to other multivariate techniques. The results of this study may be useful for policy planners and other stakeholders in making informed judgments about how to set up domestic and international trade, distribution, storage, and procurement, as well as how to manage adequate inventories ahead of time. In the future study, the system based on crop growth simulation models may be used to forecast the crop yield based on meteorological parameters.

## Data Availability

The datasets used and/or analyzed during the current study available from the corresponding author on reasonable request.
